# A novel nomogram for identifying high-risk patients among active surveillance candidates with papillary thyroid microcarcinoma

**DOI:** 10.3389/fendo.2023.1185327

**Published:** 2023-09-15

**Authors:** Li Zhang, Peisong Wang, Kaixuan Li, Shuai Xue

**Affiliations:** ^1^ Department of Nephrology, The First Hospital of Jilin University, Changchun, China; ^2^ General Surgery Center, Department of Thyroid Surgery, The First Hospital of Jilin University, Changchun, China

**Keywords:** active surveillance, papillary thyroid microcarcinoma, high risk, high-volume lymph node metastasis, extrathyroidal invasion, aggressive variant, predictive model, nomogram

## Abstract

**Objective:**

Active surveillance (AS) has been recommended as the first-line treatment strategy for low-risk (LR) papillary thyroid microcarcinoma (PTMC) according to the guidelines. However, preoperative imaging and fine-needle aspiration could not rule out a small group of patients with aggressive PTMC with large-volume lymph node micro-metastasis, extrathryoidal invasion to surrounding soft tissue, or high-grade malignancy from the AS candidates.

**Methods:**

Among 2,809 PTMC patients, 2,473 patients were enrolled in this study according to the inclusion criteria. Backward stepwise multivariate logistic regression analysis was used to filter clinical characteristics and ultrasound features to identify independent predictors of high-risk (HR) patients. A nomogram was developed and validated according to selected risk factors for the identification of an HR subgroup among “LR” PTMC patients before operation.

**Results:**

For identifying independent risk factors, multivariable logistic regression analysis was performed using the backward stepwise method and revealed that male sex [3.91 (2.58–5.92)], older age [0.94 (0.92–0.96)], largest tumor diameter [26.7 (10.57–69.22)], bilaterality [1.44 (1.01–2.3)], and multifocality [1.14 (1.01–2.26)] were independent predictors of the HR group. Based on these independent risk factors, a nomogram model was developed for predicting the probability of HR. The C index was 0.806 (95% CI, 0.765–0.847), which indicated satisfactory accuracy of the nomogram in predicting the probability of HR.

**Conclusion:**

Taken together, we developed and validated a nomogram model to predict HR of PTMC, which could be useful for patient counseling and facilitating treatment-related decision-making.

## 1 Introduction

In the past few decades, the incidence of papillary thyroid microcarcinoma (PTMC) has increased noticeably but with excellent prognosis (1). Despite its increasing incidence, the relatively stable and considerably low disease-specific mortality of PTMC in recent years has raised concerns about overdiagnosis and overtreatment (2, 3). Thus, active surveillance (AS) was recommended as an alternative disease management option for low-risk (LR) PTMC (4). According to a prospective trial of 1,235 PTMC patients in Kuma hospital in Japan, 8% of the patients had tumor enlargement ≥ 3 mm, and 3.8% demonstrated novel lymph node metastases during AS at the 10-year follow-up (5). Moreover, there were considerably fewer medical costs and unfavorable events in the AS group patients, although both immediate surgery and the AS cohorts had excellent prognosis (6). Thus, AS was chosen as the initial treatment strategy by increasing LR PTMC patients in Kuma hospital (7). Moreover, studies from other countries have also validated these results, resulting in AS being recommended as the first-line treatment strategy for LR PTMC according to the guidelines (4, 8).

Based on the patient selection criteria, LR PTMC was defined as a lack of high-grade malignancy in cytology, absence of symptoms or signs of invasion to the recurrent laryngeal nerve (RLN) or trachea, and no N1 and M1 (9). These criteria for determining AS candidature rely heavily on imaging, particularly ultrasound (US). However, preoperative imaging and fine-needle aspiration (FNA) could not rule out a small group of patients with aggressive PTMC with large-volume lymph node micro-metastasis, extrathryoidal extension (ETE) invasion to surrounding soft tissue, or high-grade malignancy from the AS candidates (10). If aggressive PTMC is considered as an “LR” category, AS would delay surgery for such patients, causing more harm than benefit. Therefore, this retrospective study was conducted to identify the preoperative predictors of high risk (HR) among “LR” PTMC patients. Then, a nomogram was developed and validated to predict the probability of HR among PTMC AS patients to facilitate preoperative decision-making.

## 2 Materials and methods

### 2.1 Study design and population

A nomogram was developed and validated according to the clinical characteristics and US features for the identification of an HR subgroup among “LR” PTMC patients before operation.

We retrospectively reviewed a total of 2,809 consecutive PTMC patients, who had undergone surgery at the Department of Thyroid Surgery, General Surgery Center at the First Hospital of Jilin University, between January 2021 and January 2022. The study was approved by the ethics committee of the First Hospital of Jilin University. The inclusion criteria were (1) PTMC confirmed by pathologic examination; (2) lobectomy (LT) with ipsilateral or total thyroidectomy (TT) with bilateral central lymph node dissection (CLND); (3) patients who were considered “LR” according to the AS selection criteria; (4) no history of neck radiation; (5) no history of thyroid surgery; and (6) the availability of complete medical records.

**Figure 1:**
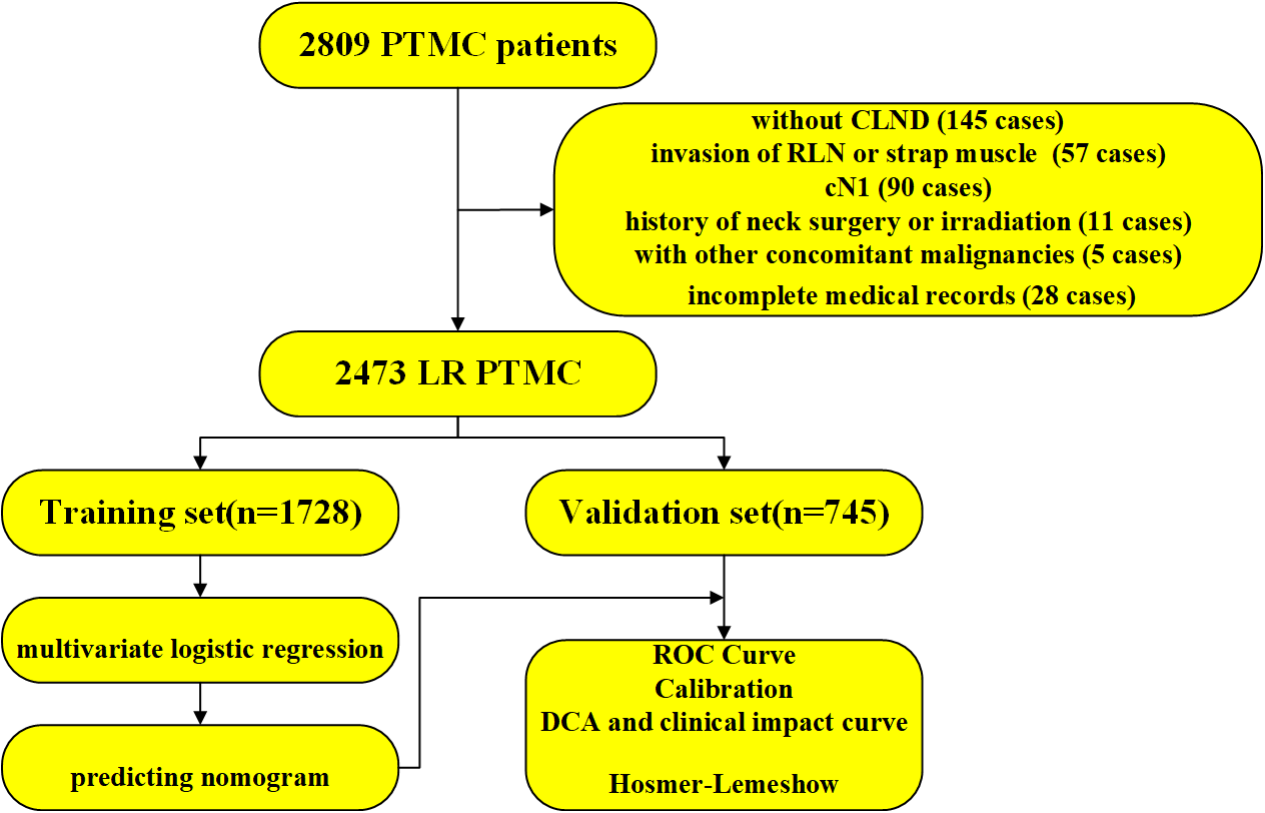
The workflow diagram of this study. PTMC, papillary thyroid microcarcinoma; CLND, central lymph node dissection; RLN, recurrent laryngeal nerve; LR, low risk; ROC, receiver operating characteristic curve; DCA, decision curve analysis.

Among 2,809 PTMC patients, 2,473 patients were enrolled in this study and 336 patients were excluded as shown in **Figure 1**. According to the chronological order of enrollment, the included participants were divided into two groups in a ratio of 7:3—a training set consisting of 1,728 consecutive patients between January 2021 and August 2021 and a validation set consisting of 745 consecutive patients between September 2021 and January 2022.

### 2.2 Surgery and variable assessment

Routine US examination with a high-resolution US scanner was performed for thyroid nodules and cervical lymph nodes in all patients. The US images were reviewed independently by three experienced US physicians. In case of disagreement, the final decision was made after discussion with another US physician with more than 10 years of experience in neck US. As previously described (11), FNA of the thyroid was recommended if the maximum diameter of a nodule was larger than 5 mm and the results of ultrasonography were suspicious. If the thyroid nodule was <5 mm in diameter, the doctor explained the risks and benefits of surgery and a decision was made according to the patient’s preference. For US-suspicious thyroid nodules or lymph nodes, FNA with or without thyroglobulin (Tg) washout was recommended for further assessment. Pathologic specimens were reviewed independently by two pathologists, and a few discordant cases were discussed with the third experienced pathologist. Regarding the surgical methods, LT was suggested for PTMC patients without any other HR factors such as gross ETE, vascular invasion, aggressive histology, and clinical N1. Otherwise, TT was performed. Patients with bilateral thyroid microcarcinoma underwent routine TT and bilateral CLND operations; CLND was performed as previously described (11).

Clinical characteristics and US features were analyzed to identify the predictive factors for HR patients: age, gender, largest tumor diameter (LTD), tumor location, echogenicity, boundary, shape, aspect ratio, calcification, CDFI, multifocality, bilaterality, and Hashimoto’s thyroiditis (HT). US features were confirmed by ultrasound for solitary tumors or the largest tumor in multifocal cases prior to surgery. Multifocality meant more than one foci of a tumor in a single lobe. Echogenicity implied both hypoechoic and nonhypoechoic masses (including isoechoic and hyperechoic). Calcification could mean no, microcalcification, macrocalcification, or both microcalcification and macrocalcification in one tumor at the same time. Blood flow features of tumors detected through Color Doppler US were divided into no (avascularity), rare (limited vascularity), and abundant (peripheral or strip-like vascularity). The diagnosis of HT was based on thyroid function examination and US: positivity for antithyroid peroxidase antibody and/or antiTg antibody and a diffusely enlarged thyroid gland with a heterogeneous echotexture on US.

### 2.3 Statistical analysis

Categorical variables were expressed as frequency and percentage (%), and continuous variables were shown as median and interquartile range. The chi-squared test was used for comparing categorical variables and the nonparametric rank sum test for comparing continuous variables. Backward stepwise multivariate logistic regression analysis was used to filter all variables to identify independent predictors of HR. Then, the nomogram was developed according to the independent predictors. Finally, a calibration curve, a concordance index (C-index), and a receiver operating characteristic (ROC) curve were constructed to assess the predictive performance of the nomogram. Moreover, decision curve analysis (DCA) and clinical impact curves were drawn to evaluate the clinical usefulness of the predictive model. The Hosmer–Lemeshow test was performed for assessing the goodness-of-fit of the model. Two-sided *p*-values < 0.05 were considered statistically significant. Statistical analyses were performed using R software (version 4.2.2).

## 3 Results

### 3.1 Patient characteristics

The study population in the training set consisted of 1,728 PTMC candidates for AS. These included 303 (17.5%) men and 1,425 (82.5%) women; the median age was 46 (38–52) years. The median tumor diameter was 0.5 (0.4–0.7) cm. All patients were considered to be in the LR group according to imaging and FNA preoperatively, while 115 (6.7%) patients were confirmed to be HR. The baseline clinical characteristics and US features in the training set are summarized in **Table 1** and show significant differences in age, sex, LTD, multifocality, and bilaterality between the LR and HR groups.

**Table 1:** Baseline characteristics and risk factors of the high-risk group in the derivation cohort.

Characteristics	Total	LR	HR	*p*-value
Total, *n* (%)	1,728 (100)	1,613 (93.3)	115 (6.7)	
Age, median [IQR]	46.00 [38.00, 52.00]	46.00 [38.00, 52.00]	39.00 [32.00, 47.50]	<0.001
Gender, *n* (%)				<0.001
Male Female	303 (17.5) 1,425 (82.5)	253 (15.7) 1,360 (84.3)	50 (43.5) 65 (56.5)	
LTD, median [IQR]	0.50 [0.40, 0.70]	0.50 [0.40, 0.70]	0.70 [0.55, 0.80]	<0.001
Tumor location, *n* (%)				0.044
Upper Middle Lower Isthmus	422 (24.4) 622 (36.0) 615 (35.6) 69 (4.0)	406 (25.2) 571 (35.4) 572 (35.5) 64 (4.0)	16 (13.9) 51 (44.3) 43 (37.4) 5 (4.3)	
Echogenicity, *n* (%)				1.000
Nonhypoechoic Hypoechoic	29 (1.7) 1,699 (98.3)	27 (1.7) 1,586 (98.3)	2 (1.7) 113 (98.3)	
Boundary, *n* (%)				0.728
Not clear Clear	1,633 (94.5) 95 (5.5)	90 (5.6) 1,523 (94.4)	5 (4.3) 110 (95.7)	
Shape, *n* (%)				0.804
Not regular Regular	1,581 (91.5) 147 (8.5)	1,477 (91.6) 136 (8.4)	104 (90.4) 11 (9.6)	
Aspect ratio, *n* (%)				0.129
≤1 >1	630 (36.5) 1,098 (63.5)	580 (36.0) 1,033 (64.0)	50 (43.5) 65 (56.5)	
Calcification, *n* (%)				<0.001
No Micro Macro Both	883 (51.1) 731 (42.3) 99 (5.7) 15 (0.9)	849 (52.6) 658 (40.8) 93 (5.8) 13 (0.8)	34 (29.6) 73 (63.5) 6 (5.2) 2 (1.7)	
CDFI, *n* (%)				0.022
No Rare Abundant	850 (49.2) 573 (33.2) 305 (17.7)	807 (50.0) 523 (32.4) 283 (17.5)	43 (37.4) 50 (43.5) 22 (19.1)	
Multifocality, *n* (%)				0.011
No Yes	1,128 (65.3) 600 (34.7)	1,066 (66.1) 547 (33.9)	62 (53.9) 53 (46.1)	
Bilateral, *n* (%)				0.010
No Yes	1,280 (74.1) 448 (25.9)	1,207 (74.8) 406 (25.2)	73 (63.5) 42 (36.5)	
HT, *n* (%)				0.239
No Yes	1,169 (67.7) 559 (32.3)	1,085 (67.3) 528 (32.7)	84 (73.0) 31 (27.0)	

LR, low risk; MHR, medium-high risk; LTD, largest tumor diameter; HT, Hashimoto’s thyroiditis.

### 3.2 Development of the nomogram model

**Figure 2:**
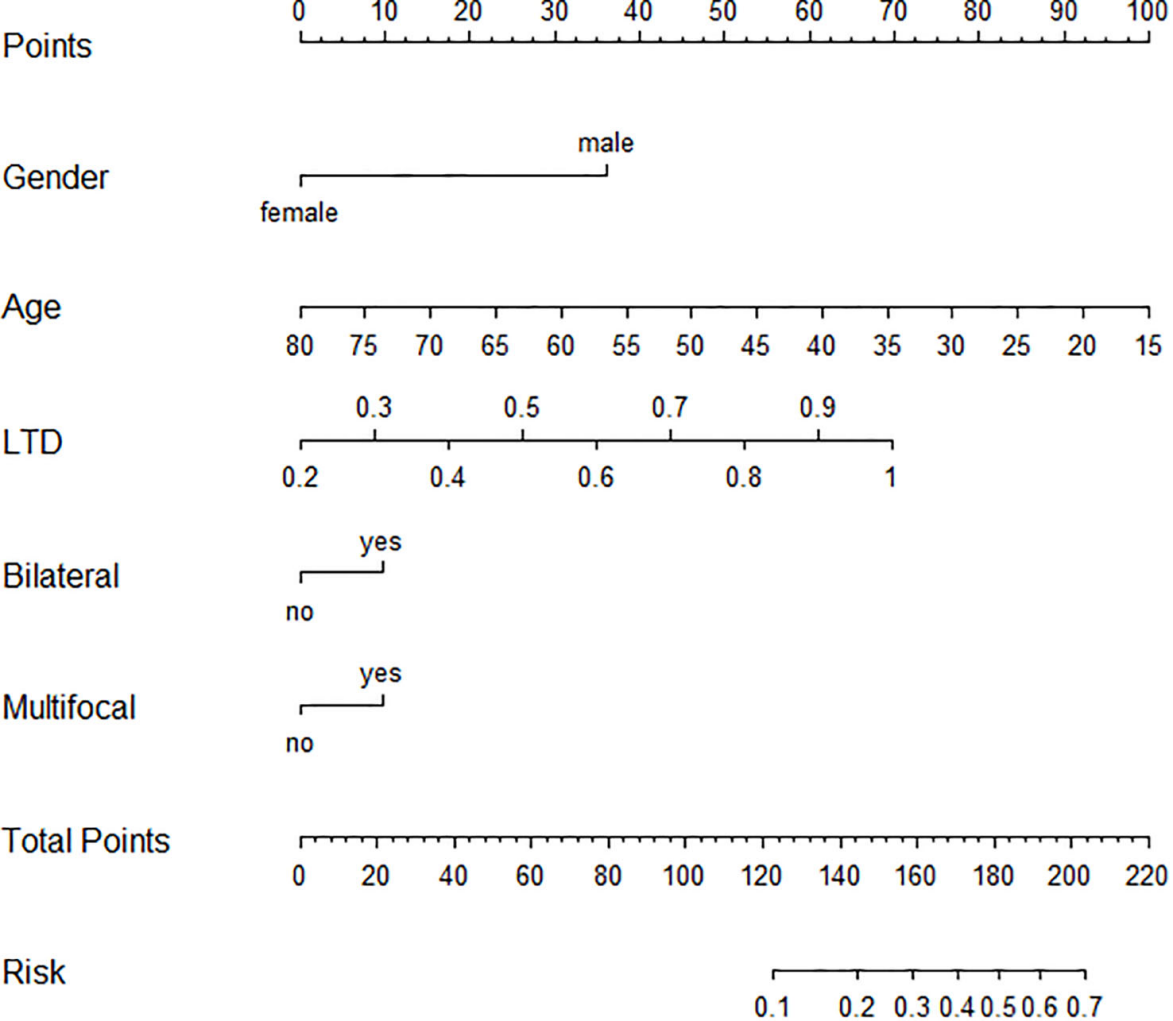
Graphic nomogram based on a multivariable logistic regression model for prediction of HR patients among AS candidates. HR, high risk; AS, active surveillance.

For identifying independent risk factors, multivariable logistic regression analysis was performed using the backward stepwise method and revealed that male sex [3.91 (2.58–5.92)], older age [0.94 (0.92–0.96)], LTD [26.7 (10.57–69.22)], bilaterality [1.44 (1.01–2.3)], and multifocality [1.14 (1.01–2.26)] were independent predictors of the HR group (**Table 2**). Based on these independent risk factors, a nomogram model was developed for predicting the probability of HR (**Figure 2**). The baseline characteristics and predictors for HR in the validation cohort are given in **Supplementary Appendix SA1**.

**Table 2:** Multivariable logistic regression analysis for predictive factors of the medium- to high-risk group in the derivation cohort.

	Odds ratio [95% CI]	p-value
Gender: Male	3.91 [2.58, 5.92]	<0.001
Age	0.94 [0.92, 0.96]	<0.001
LTD	26.7 [10.57, 69.22]	<0.001
Bilateral: Yes	1.44 [1.01, 2.30]	0.03
Multifocal: Yes	1.14 [1.01, 2.26]	0.04

LTD, largest tumor diameter.

### 3.3 Validation of the nomogram model

**Figure 3:**
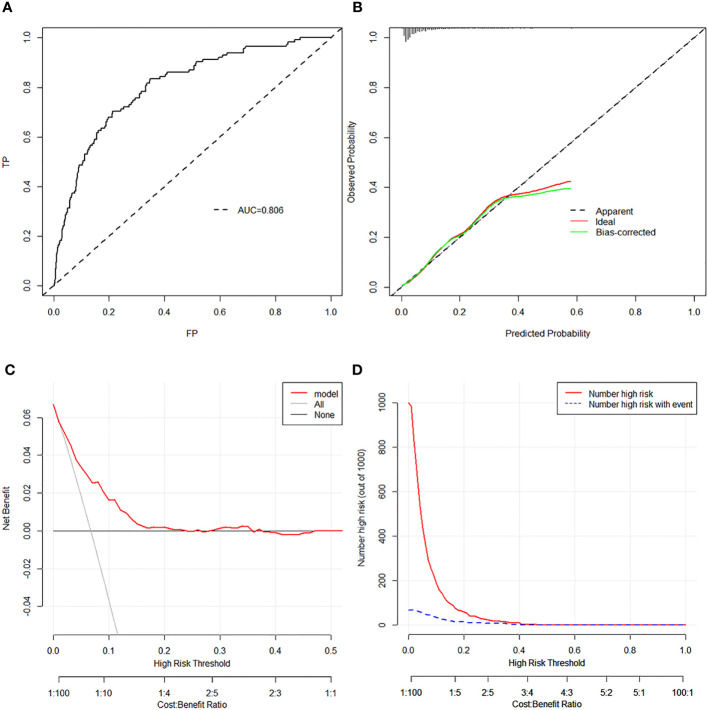
**(A)** The ROC analysis with AUC for training set. **(B)** Discrimination plot for training set. **(C)** DCA revealed that preoperative “LR” PTMC patients would benefit more if this model was used to predict the risk of HR when the threshold probability is between >1% and <20% in the training set. **(D)** The clinical impact curve of the training set revealed that the patients categorized as HR by the nomogram were more likely to be truly HR when the threshold probability < 40%. ROC, receiver operating characteristic curve; AUC, area under curve; DCA, decision curve analysis; LR, low risk; PTMC, papillary thyroid microcarcinoma; HR, high risk.

Bootstrap validation was performed for the internal validation of the nomogram. The C index was 0.806 (95% CI, 0.765–0.847), which indicated satisfactory accuracy of the nomogram in predicting the probability of HR, also shown as the ROC curve (**Figure 3A**). The calibration curve demonstrated good agreement between the probability predicted by the model and the actual incidence of HR (mean absolute error: 0.016) (**Figure 3B**). Moreover, both DCA and clinical impact curves were used to assess the clinical utility of the nomogram. The DCA curve indicated that preoperative “LR” PTMC patients would benefit more if this model was used to predict the risk of HR when the threshold probability is between >1% and <20% (**Figure 3C**). The clinical impact curve (**Figure 3D**) indicated that the patients categorized as HR by the nomogram were more likely to be truly HR when the threshold probability < 40%. Additionally, the good calibration of the model was evaluated by the Hosmer–Lemeshow test (*p* = 0.243). The Brier score of 0.056 showed a good overall performance.

In the validation cohort, the calibration, ROC curve, Hosmer–Lemeshow test, DCA curve, and clinical impact curve, given in **Supplementary Appendix SA2**, also showed good overall performance.

## 4 Discussion

To our knowledge, this is the first study to identify factors predicting HR in “LR” PTMC patients based on preoperative examination. In our “LR” PTMC cohort, 170 of the 2,473 (6.9%) patients were reclassified into the HR group after surgery based on the results of the pathologic examination. Overall, 170 PTMCs were categorized into the HR group based on the presence of the following: more than five micro-lymph node metastases (95 cases), soft tissue invasion (64 cases), vascular invasion (2 cases), RLN invasion (2 cases), aggressive variant (1 case), and two more risk factors (6 cases). Multivariant regression analysis found male sex, younger age, larger tumor size, bilaterality, and multifocality to be independent predictors for the HR group. Moreover, the nomogram model was constructed and validated using the training and validation sets.

Gross ETE, or macroscopic ETE, was a risk factor for recurrence and disease-specific mortality (12). Thus, PTMCs with gross ETE should be excluded from the AS cohort at the time of patient selection, rendering the imaging of PTMC, especially US, extremely important for the diagnosis of gross ETE. As shown in **Figure 1**, 32 cases of gross ETE to strap muscle were all diagnosed correctly, and 23 cases (23/25, 92%) of gross ETE to RLN were confirmed before surgery. Strap muscle invasion is much easier to diagnose because of the obviously different echogenicity between thyroid cancer and muscle (13). There are few reports of RLN invasion by PTMC because of the low incidence. In Ito’s study, only 9 of 1,143 PTMC patients were diagnosed with RLN invasion (14). As all tumor diameters with RLN invasion were 7 mm or larger, it can be concluded that tumors of <7 mm are not likely to invade the RLN (14). According to data from Memorial Sloan Kettering Cancer Center, gross invasion of the RLN was not observed for tumors of <9 mm in diameter, regardless of tumor location (15). In our study, only 25 of 2,809 patients were diagnosed with RLN invasion, an incidence similar to that reported by Ito et al. However, the tumor diameters of two patients with RLN invasion were lower than 7 mm (5 mm and 6 mm), and two cases with RLN invasion were not differentiated correctly because the coarse calcification of the tumor hindered boundary evaluation. Microscopic invasion of the soft tissue of the tumor was also considered a risk factor for recurrence, although reports of PTMC with microscopic invasion to soft tissue are rare (16). Whether minimal invasion into the soft tissue plays an important role in the prognosis of PTMC is still unknown. In 2019, in a BRAFV600E-mutated multifocal PTMC cohort, we had found that the recurrence rate of ETE to soft tissue was 6.6% while that of intrathyroidal PTMC was 3.8% (11). These patients were probably not differentiated before surgery due to the greater difficulty in diagnosing microscopic invasion to soft tissue using US. Moon et al. established a sonographic T staging method to predict the ETE degree of PTMC and found that it had high sensitivity and specificity (17); however, this method was not validated in other studies.

Although preoperative diagnosis is very difficult for high-volume central lymph node metastasis (CLNM) in cN0 PTMC, the incidence is low, ranging from 3.8% to 9.5% (18–20). In our “LR” PTMC patients, 3.8% (95/2,473) of the patients were diagnosed as high-volume CLNM. Moreover, studies have shown that younger age, male sex, tumor size > 5 mm, ETE, multifocality, microcalcification, capsular invasion, and abundant blood flow are independent clinicopathological risk factors for high-volume CLNM in cN0 PTMC patients (21–23), which in agreement with our results. Furthermore, the incidence of aggressive variants is low. According to the American National Cancer Database from 2004 to 2015, among 83,198 cases of PTMC, the classic variant accounted for 98.6%, the tall-cell variant (TCV) accounted for 1.1%, and diffuse sclerosis accounted for 0.3% of all PTMC cases (24). In another study of 745 cases of PTMC, only 1 (0.13%) patient was diagnosed with the hobnail variant (25). Although the aggressive variant is associated with pathologic features exhibiting greater invasion, there were no differences in overall survival of patients with this variant compared with that of patients with classical PTMC after treatment (25–27). Additionally, studies have shown some US features in TCV-PTMC, for example, a hypoechoic halo around nodules and hypoechoic nodules with a localized central isoechoic lesion were much more common in TCV-PTMC (28). Moreover, TCV-PTMC showed nodules with a more regular margin and less microcalcification than classical PTMC (29). In our study, only one patient was diagnosed with the TCV after surgery. Vascular invasion of the tumor is typically associated with a poor prognosis. However, studies on vascular invasion of PTMC are scarce, and only two patients showed vascular invasion in this study, indicating that the incidence of vascular invasion in PTMC is probably low.

When PTMC patients choose between AS and surgery, they would like to know the possibility of HR after surgery. Without reliable molecular markers and an accurate imaging method, a small group of HR PTMC patients will be misdiagnosed as “LR”, which will be harmful for the patients. To avoid this, we identified preoperative factors, such as younger age, male sex, larger tumors, bilaterality, and multifocality, to establish a nomogram for predicting the possibility of HR in our study. Younger age has been proven to be an important factor for disease progression during AS (30). Mounting evidence also confirmed that younger age, male sex, larger tumors, bilaterality, and multifocality were related to a greater degree of CLNM and a higher recurrence rate (18, 19, 21). Studies have provided strong evidence that bilateral multifocality, rather than unilateral multifocality, should be considered an aggressive marker of PTMC (31, 32). In another study, when unilateral multifocality and bilaterality coexisted in PTMC, patients had the highest risk of CLNM and possibly of local recurrence compared with those with either risk factor alone (31). Based on these factors, we could differentiate some HR PTMC patients from “LR” patients and recommend surgery as soon as possible.

There are several limitations in our study. First, this is a retrospective single‐center study, which could have probably given rise to selection biases despite the large sample size. Furthermore, molecular markers, such as BRAF and TERT, were not considered in our study, which might affect the risk category. Finally, only the internal validation method was used for developing the nomogram in this study. Therefore, prospective multi-center studies, especially considering molecular markers, should be conducted to obtain more objective conclusions.

In summary, the predictors of the HR subgroup in the AS candidates of PTMC patients were male sex, younger age, larger tumor size, bilaterality, and multifocality. Based on preoperative clinical and US factors, we developed and validated a nomogram model to predict HR of PTMC, which could be useful for patient counseling and facilitating treatment-related decision-making.

## Data availability statement

The raw data supporting the conclusions of this article will be made available by the authors, without undue reservation.

## Ethics statement

The studies involving human participants were reviewed and approved by the ethics committee of the First Hospital of Jilin University. Written informed consent to participate in this study was provided by the participants’ legal guardian/next of kin.

## Author contributions

All authors listed have made a substantial, direct, and intellectual contribution to the work, and approved it for publication.
