# Supplementary material for: A novel nomogram for identifying high-risk patients among active surveillance candidates with papillary thyroid microcarcinoma
Source: Front Endocrinol (Lausanne). 2023 Sep 15;14:1185327. doi: 10.3389/fendo.2023.1185327 (PMC10541211; doi:10.3389/fendo.2023.1185327)
Supplement: Supplementary file 1 [file Presentation_1.zip › SA2.docx]

**Figure legend:**

**Figure SA2**：(A) The ROC analysis with AUC for validation set. (B) Discrimination plot for validation set. (C) DCA revealed that preoperative “LR” PTMC patients would benefit more if this model was used to predict the risk of HR when the threshold probability is between >1% and <20% in the validation set. (D) The clinical impact curve of the validation set revealed that the patients categorized as HR by the nomogram were more likely to be truly HR when the threshold probability < 40%. ROC:receiver operating characteristic curve; AUC: area under curve; DCA: decision curve analysis; LR:low risk; PTMC:papillary thyroid microcarcinoma; HR:high risk.
