# Supplementary material for: A novel nomogram for identifying high-risk patients among active surveillance candidates with papillary thyroid microcarcinoma
Source: Front Endocrinol (Lausanne). 2023 Sep 15;14:1185327. doi: 10.3389/fendo.2023.1185327 (PMC10541211; doi:10.3389/fendo.2023.1185327)
Supplement: Supplementary file 1 [file Presentation_1.zip › SA1.docx]

Table SA1. Baseline Characteristics and Risk Factors of Medium-High Risk Group in the Validation Cohort

| **Characteristics** | **Total** | **LR** | **MHR** | **p-Value** |
| --- | --- | --- | --- | --- |
| Total,n (%) | 745(100) | 690(92.6) | 55(7.4) |  |
| Age ,median [IQR] | 45.00 [37.00, 52.00] | 45.00 [38.00, 52.00] | 39.00 [32.00, 47.00] | <0.001 |
| Gender,n (%)  Male  Female | 156 (20.9)  589 (79.1) | 126 (18.3)  564 (81.7) | 30 (54.5)  25 (45.5) | <0.001 |
| LTD ,median [IQR] | 0.50 [0.30, 0.70] | 0.50 [0.30, 0.70] | 0.70 [0.50, 0.80] | <0.001 |
| Tumor Location,n (%)  Upper  Middle  Lower  Isthmus | 182 (24.4)  266 (35.7)  266 (35.7)  31 ( 4.2) | 171(24.8) 243(35.2) 246(35.7) 30 ( 4.3) | 11 (20.0)  23 (41.8)  20 (36.4)  1 ( 1.8) | 0.596 |
| Echogenicity,n (%)  Nonhypoechoic  Hypoechoic | 11 ( 1.5)  734 (98.5) | 10 ( 1.4) 680 (98.6) | 1 ( 1.8)  54 (98.2) | 1.000 |
| Boundary,n (%)  Not clear  Clear | 713 (95.7)  32 ( 4.3) | 660 ( 95.7) 30 (4.3) | 53 ( 96.4)  2 (3.6) | 1.000 |
| Shape,n (%)  Not regular  Regular | 697 (93.6)  48 ( 6.4) | 647 ( 93.8) 43 (6.2) | 50 ( 90.9)  5 (9.1) | 0.585 |
| Aspect Ratio,n (%)  ≤1  ＞1 | 259 (34.8)  486 (65.2) | 237 (34.3) 453 (65.7) | 22 (40.0)  33 (60.0) | 0.484 |
| Calcification,n (%)  No  Micro  Macro  Both | 402 (54.0)  275 (36.9)  56 ( 7.5)  12 ( 1.6) | 381 (55.2) 246 (35.7) 54 ( 7.8) 9 ( 1.3) | 21 (38.2)  29 (52.7)  2 ( 3.6)  3 (5.5) | 0.004 |
| CDFI,n (%)  No  Rare  Abundant | 372 (49.9)  261 (35.0)  112 (15.0) | 346 (50.1) 239 (34.6) 105 (15.2) | 26 (47.3)  22 (40.0)  7 (12.7) | 0.700 |
| Multifocality,n (%)  No  Yes | 503 (67.5)  242 (32.5) | 479 (69.4) 211 (30.6) | 24 (43.6)  31 (56.4) | 0.001 |
| Bilateral,n (%)  No  Yes | 561 (75.3)  184 (24.7) | 532 (77.1) 158 (22.9) | 29 (52.7)  26 (47.3) | 0.001 |
| HT,n (%)  No  Yes | 518 (69.5)  227 (30.5) | 475 (68.8) 215 (31.2) | 43 (78.2)  12 (21.8) | 0.195 |

LR:low-risk, MHR:medium-high risk,LTD:largest tumor diameter,HT:Hashimoto’s Thyroiditis

Table 2. Multivariable Logistic Regression Analysis for Predictive Factors of Medium-High Risk Group in in the Derivation Cohort

|  | Odds ratio [95% CI] | p-Value |
| --- | --- | --- |
| Gender:Male | 5.96 [3.25 11.08] | <0.001 |
| Age | 0.96 [0.92 0.99] | <0.001 |
| LTD | 15.2 [3.97 61.34] | <0.001 |
| Bilateral: Yes | 1.97 [1.01 3.82] | 0.04 |
| Multifocal: Yes | 2.31 [1.21 4.44] | 0.01 |

LTD:largest tumor diameter
