# Supplementary figures and images for: A novel nomogram for identifying high-risk patients among active surveillance candidates with papillary thyroid microcarcinoma
Source: Front Endocrinol (Lausanne). 2023 Sep 15;14:1185327. doi: 10.3389/fendo.2023.1185327 (PMC10541211; doi:10.3389/fendo.2023.1185327)

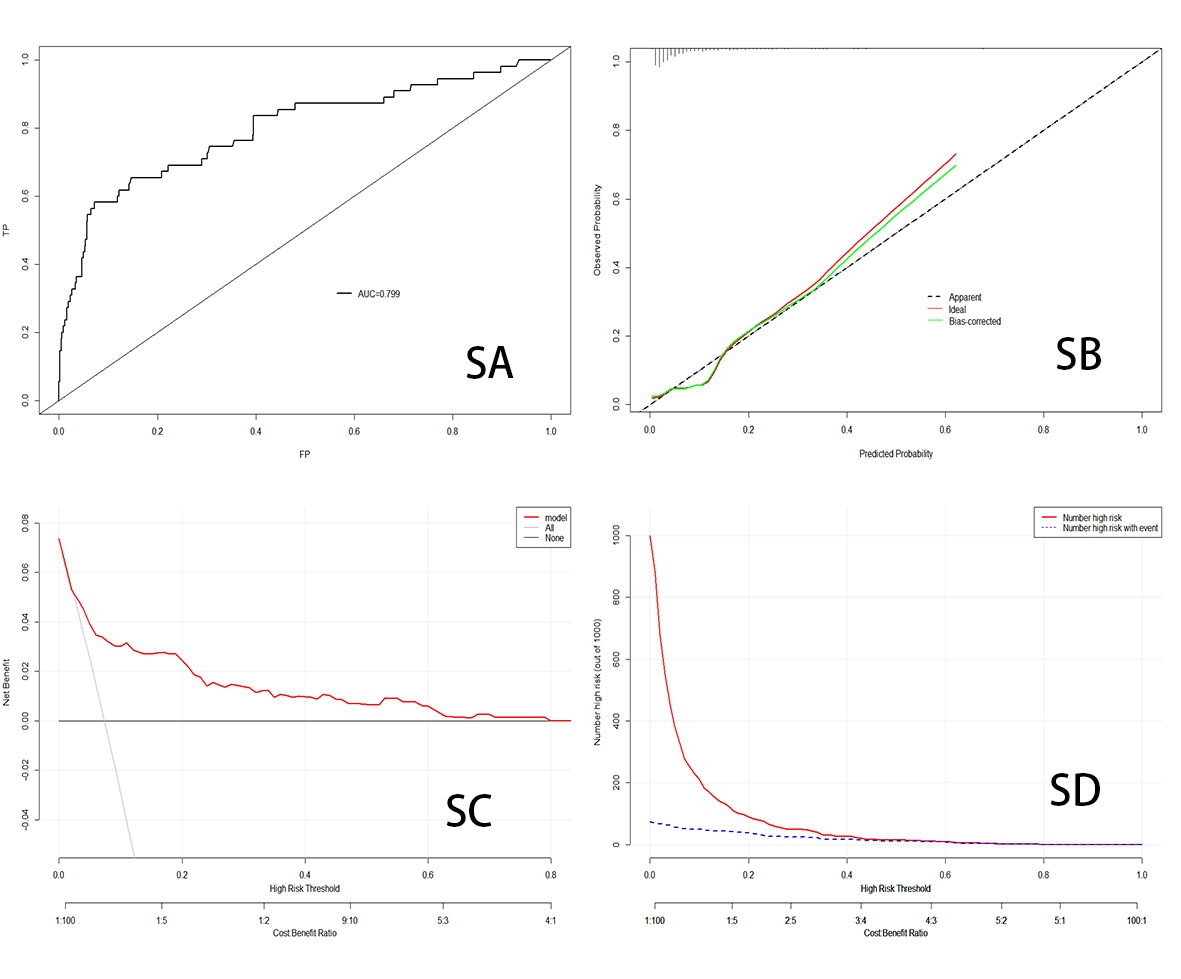

Supplement: Supplementary file 1 [file Presentation_1.zip › SA2.tif]
